# The function(s) of consciousness revisited: insights from a modular/constitutive model using vision as a test case

**DOI:** 10.3389/fpsyg.2026.1829430

**Published:** 2026-06-22

**Authors:** Thurston Lacalli

**Affiliations:** Department of Biology, University of Victoria, Victoria, BC, Canada

**Keywords:** conscious vision, gaze control, memory theories of consciousness, position-dependence, the hard problem

## Abstract

This paper extends an earlier analysis of the functions attributable to consciousness, applying it to a modular/constitutive model that limits the role conscious sensations are allowed to play in integrative processes operating at scale. The focus is on conscious vision because of the challenge it poses for any theory of consciousness, of how to account for a 2-dimensional perceptual display. For a modular/constitutive model, what is required is that awareness of visual stimuli has the property of position-dependence, meaning that the physical location of each module contributing to the visual experience has perceptual consequences. Conscious gaze control can then act as a device for registering salient visual features in a way that has no preconscious counterpart, while contributing also to the process by which visual experience acquires noetic content. The analysis provides (1) theoretical justification for Merker’s ideas on vision and gaze control as a way of understanding the core functions consciousness performs for our species, where the post-natal period during which visual skills are refined equates to the learning processes through which agency is acquired, and (2) a framework for thinking about the hard problem and the adaptive advantage conscious vision confers over all possible zombie alternatives. This would imply that conscious experience for species that rely either less than we do on vision or not at all may nevertheless incorporate a position-dependent component, which for those species either could or would depend on something other than light perception. Hence the argument is not that conscious vision was necessarily a component of consciousness at it first evolved, but that the contrasting roles position-dependent contents play in behavioral control compared with those conferring valence requires that both be present if the adaptive advantage of an emergent consciousness is to depend on its ability to assign meaning to experience. This leaves by default a model of proximate cause where it is the combined advantages of conscious attention control and memory encoding that makes consciousness adaptive, and a minimalist conception of the functions performed by consciousness if it is not a direct participant in large-scale integrative processes.

## 1 Introduction

This is one of a series of papers examining consciousness from an evolutionary perspective, the intent in this installment being to extend my analysis of the functions attributable to consciousness (Lacalli, 2024b) to the modular/constitutive model of consciousness introduced in the paper immediately preceding this one (Lacalli, 2025)^1^. The central premise of the model is that conscious sensations are generated by groups of neurons (the modules) that can be small in relative terms and localized elsewhere than the cortex. Evidence for this comes from the work of Berridge and Kringelbach (2015) on the sub-cortical hotspots responsible for sensations of pleasure (of liking rather than wanting), which begs the question of whether sensations generated locally might also be restricted to having only local effects. This would add an additional constraint to the way such a consciousness would function and, for a broadcast model of consciousness dependent on a signal, makes explicit the problem of signal range, of whether it is large or small, and how the signal is propagated, or if it is. The point here is that few theories pay much attention to the response component of conscious awareness, that a broadcast signal only has causal effects if it is monitored and responded to (see Irwin, 2023 on the monitor function). If this depends on comparatively small groups of neurons that play no direct role in integrative processes operating on a large scale, there would be no route by which conscious sensations could do so either. Hence, though a minority view (Merker, 2007; Earl, 2019), it remains within the bounds of possibility that integrative processes coordinated by the cortex operate largely if not exclusively in an unconscious mode. And while this is at odds with intuition, that consciousness is “big” (Blumenfeld, 2023) and integrative on a large scale (see Morsella et al., 2016 on the integrative consensus), the alternative, that sensations are produced and act locally, and only locally, cannot be ruled out on scientific grounds (Lacalli, 2025). The intuitive sense that consciousness is both big and integrative would then be explained simply as a sampling bias: that we are consciously aware of no more than a fraction of the brain’s operations, which loom large in our imagination only because they are conscious.

The modular/constitutive model of consciousness then invites further investigation, my intent here being to demonstrate its utility as a conceptual device for thinking about the functions consciousness evolved to perform, including the minimum it must do to be adaptive if it does not participate directly in large-scale integrative processes. First, in section 2, there are theoretical issues requiring attention, among which is the problem of explaining the adaptive advantage of consciousness if, as proposed by Polák and Marvan (2019), non-conscious phenomenal states could perform many of its functions just as effectively. There is also a preconception to be dealt with, that the evolution of consciousness was simply a matter of converting neural pathways operating in a non-conscious mode to a conscious one. The alternative, the premise of the modular/constitutive model, is that the production of conscious sensations depends on neural structures and circuits that have no preconscious counterparts operating in a fashion that also has no preconscious counterpart. Section 3 is devoted to conscious vision, both how a 2-dimensional percept might be constructed, but also what is required for conscious vision to have an advantage over unconscious visual search. This leads to conclusions that largely validate the ideas of Merker (2012, 2013) on gaze control as the key to understanding the adaptive advantage of conscious vision, providing theoretical support for his analysis. Section 4 explores the learning task required if Cleeremans’ premise is correct, that consciousness must be learned (Cleeremans, 2011; Cleeremans et al., 2020) or, more precisely, that if conscious agency depends on a learning process, what that process is.

A crucial part of the argument in sections 3 and 4 depends on experiential states of awareness having ***properties***. The term is intended here to refer to features intrinsic to such states that are more fundamental than those more routinely used to describe the qualitative character of conscious experience, when constructing similarity or quality spaces for example (Raffman, 2015; Rosenthal, 2015), or in my own analysis of experience space (Lacalli, 2021, 2022). These more fundamental properties must then exhibit features unique to consciousness and guide behavior in ways that cannot be duplicated by non-conscious mechanisms. The only obvious example I can provisionally identify, given this more stringent requirement, is position-dependence, the supposition that the position of each module in physical space has perceptual consequences. This, for vision, provides an explanation for the construction of a visual percept and the precondition required if an alternative form of gaze control is to be imposed over one previously dependent on reflex alone. There should then be mechanistic differences between conscious and reflexive forms of gaze control, where the assumption is that conscious vision would have evolved to take advantage of those differences. The argument is more complex if unconscious vision was instead converted from an unconscious mode of operation to a conscious one, but the conclusion does not change, evidence that the hard problem may not be as restrictive as usually supposed in constraining what can and cannot be said about the adaptive advantage of consciousness. A focus on gaze control differs from previous arguments that I and others have made regarding learning and memory, as central to the adaptive advantage conferred by consciousness (Bronfman et al., 2016; Jablonka and Ginsburg, 2022; Budson et al., 2022; Lacalli, 2024a,b). The issue here is not that consciousness is less important to learning and memory systems than those accounts suppose, but a recognition that the advantage of consciousness lies in the synergies it makes possible.

The analysis is extended in section 5 to deal with conscious contents other than vision, with a recognition that there are species that rely less on vision than we do, nocturnal mammals for example and bats in particular, and taxa with an expanded sensory response, e.g., to thermal radiation in the case of pit vipers. Despite these differences, the distinction that matters for this analysis is that between contents that exhibit position-dependence, which could be vision as we experience it or something else, and those, mainly valence-related, that do not. The question at issue is whether it is possible to deduce, simply through an exercise in logic, if both would necessarily have been part of an emergent consciousness, or in contrast, if a consciousness consisting of contents belonging to just one category, say just vision, or just affect, would be possible. At its core this is a question about whether the reinforcement process required to learn agency must be conscious, which equates to asking whether the adaptive advantage of consciousness depends on its ability to assign meaning to sensory experience. Ideas are advanced to explain why valence, and hence meaning, evolved, but the analysis falls short of answering either that question or what I have referred to as Velmans’ question (see Velmans, 2012), as to the proximate reason consciousness itself evolved. This limits what one can say about the sequence in which contents were added to an evolving consciousness, but brings more clearly into focus the questions that need answers if we are to discover what that sequence was.

## 2 Conceptual issues: causal efficacy and adaptive advantage, conversion versus reallocation

My formulation of the problem posed by consciousness follows that introduced in the paper preceding this one (Lacalli, 2025), that to understand consciousness at a foundational level two things must be explained: (1) existence, the ontological question of how consciousness can exist at all, and (2) realization, of how consciousness comes to be realized by biological brains. For a broadcast model of consciousness, these are equivalent to asking first, how a signal can exist that a biological brain can respond to by generating a state of subjective awareness, and second, how awareness is achieved by that brain at a mechanistic level. But defining awareness in this way lodges it firmly in the hard problem, which is generally assumed to prevent us from making mechanistic assumptions regarding its nature and physical properties. Beyond these two foundational questions, there are two further problems to consider that relate to evolutionary concerns more specifically, of (3) the adaptive advantage consciousness confers over non-conscious neural processes, and (4) why consciousness is not epiphenomenal, which it cannot be if its existence is to be explained in evolutionary terms (Lindahl, 1997). As to how these four questions are related, my assessment of the situation is that the first two, in accord with Chalmers (1995), remain fundamental issues that no theory has yet adequately addressed, whereas the last two depend on theoretical stance and may potentially be answerable. The remainder of this analysis is designed to show how, for question (3) in particular, this may be possible.

**FIGURE 1:**
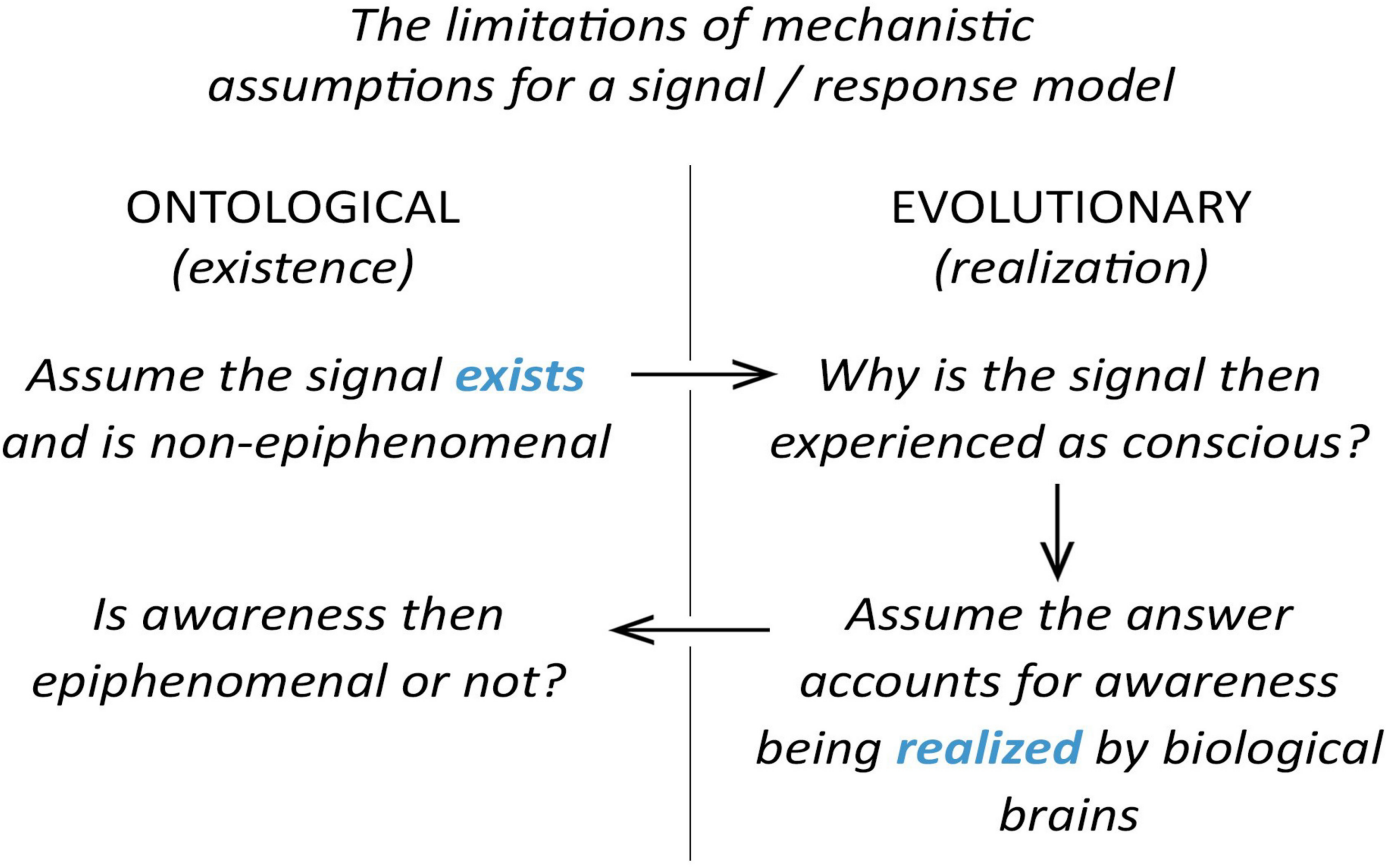
Mental causation for a broadcast (signal/response) model of consciousness in two time scales: in real time the ontological issue of causal efficacy, and in evolutionary time the problem of why those effects confer an adaptive advantage. The conundrum this illustrates arises from the hard problem, that assumptions about the causal efficacy of a mechanistic signal say nothing about whether awareness of the signal is also causally efficacious. The cycle can be broken at two points, but doing so on the ontological side depends on understanding the nature of consciousness as a component of physical reality, a daunting task, while on the evolutionary side it depends on identifying the adaptive advantage of consciousness over all competing non-conscious alternatives. The premise of this paper is that a modular/constitutive model of consciousness, combined with the property (for vision specifically) of positional dependence, may resolve the evolutionary question, thus breaking the cycle. Doing so offers the prospect of advancing our understanding of the advantages conferred by conscious vision while providing a starting point for addressing Velmans’ question, of the proximate reason consciousness evolved.

Figure 1 illustrates the conceptual conundrum represented by questions (3) and (4). Suppose we begin with a set of mechanistic assumptions about the source of consciousness modeled as a broadcast signal. If we assume in addition that this signal is non-epiphenomenal, a scenario can be constructed whereby consciousness *could* evolve except that, without knowing if consciousness has any adaptive advantage, we have no explanation for why it *would* have evolved. For an EM field-based theory, the point is easily illustrated: that conscious sensations are for those theories assumed to depend on solutions to field equations, but this does not explain why the effects specified by those solutions are more adaptive when perceived consciously. The message here accords with Chalmers’ view, that mechanistic assumptions deliver no insights when it comes to solving the hard problem, but also no insights into the adaptive advantage consciousness confers over having the brain operate in the dark, without consciousness. Hence, however detailed our knowledge concerning the mechanistic effects of a broadcast signal, that knowledge tells us nothing about the causal efficacy of our conscious awareness of that signal, thwarting any attempt to explain how the qualitative features of our awareness of different kinds of sensory input evolved as it has, generating qualia with distinctive characteristics. The logic of the argument then has a certain symmetry, that we return to our starting point none the wiser about why a consciousness consisting of particular and distinguishable experiential states evolved.

But seen through a different lens, the issue here is the same as that raised by Polák and Marvan (2019), that every consciously-perceived phenomenal state could have a non-conscious counterpart that achieves the same result without consciousness. This is the zombie argument in a different guise, which is not to suggest that a zombie equivalent of a human being (a philosophical zombie in the classical sense) could conceivably evolve, but instead, casting the argument in more mechanistic terms, that for each function one might suppose consciousness performs there could conceivably be a non-conscious mechanism, its zombie counterpart, that produces precisely the same behavioral outcome for the same metabolic cost. For phenomenal contents this is a difficult argument to refute if non-conscious phenomenal states of the kind proposed by Polák and Marvan (2019) can exist as a consequence of neural activity, and I see no reason currently to reject that possibility. But contents more complex than phenomenal ones are a different matter, meaning those with an intrinsic structure, including vision. Here a resolution of the conundrum may be possible so long as awareness has properties, a statement whose meaning and implications are examined more fully in sections 3–5^2^.

First, however, attention is required to an assumption widespread in the theoretical literature, that the evolution of consciousness is simply a matter of converting existing neural pathways from a non-conscious mode of operation to a conscious one. For theories where experiential states are supposed to depend on the activities of connectomal networks operating on a large scale, either because of their structure or the nature of the computations they perform, those structures and computations would be preserved across the transition and directly shape the way consciousness is experienced. The alternative is that there is no such continuity because consciousness owes its origin to innovations in neural structure and activity entirely separate from those on which non-conscious neural functions depend, so a mapping of structures and activity patterns from one to the other would not be expected. The evolutionary process would in consequence be less a matter of conversion than what I will refer to as reallocation: that control over a particular subset of behaviors, previously carried out reflexively, has been reallocated so as to incorporate a conscious component that has no preconscious counterpart.

Which of these options, conversion or reallocation, is the more convincing as an evolutionary scenario? Neither can be ruled out because evolution has in the past used both, a skeletal example of conversion being the progressive substitution of bone for cartilage as the vertebrate skeleton evolved. That transition largely conserved the existing pattern of cartilaginous elements, so an understanding of how and why the bony skeleton came to be structured as it is depends in large part on asking those same questions about its cartilaginous precursor. In contrast, the innovations that led to the evolution of endoskeletal support in the first place illustrate the alternative, of reallocation, in that case accompanied by a replacement of one set of structures by another. Internal skeletons were preceded, in basal deuterostomes (hemichordates and the like) by a hydraulic system of coelomic cavities and diverticula that supported the body in an entirely different way than an endoskeleton does. Once hydraulic support was no longer required, the coelomic specializations providing that support could be dispensed with resulting in a much simpler arrangement of coelomic compartments performing subordinate tasks, which implies that for cases where the evolutionary innovation employs an entirely different mechanism, reallocation may be the more likely outcome.

The issue is not then how many ways there are to explain consciousness, because there are many competing proposals, but instead the combinations possible when there are but two sets of binary possibilities as outlined above, that (1) consciousness is either big and integrative or small, modular and non-integrative and (2) that it evolved either by conversion or reallocation. This yields four options in total, that consciousness is Large and evolved by Conversion (LC), Small and evolved by Reallocation (SR) or, the reverse combination, Large and Reallocated (LR) or Small and Converted (SC). Statistically, each option has a 25% chance of being correct, but in practice it is the difference between LC and SR that most clearly mirrors the issues raised in the previous two paragraphs. Chiefly the problem is that adopting the LC option focuses attention on the output of neuro-computational processes rather than inputs to those processes, which may be a conceptual trap if the burden of conversion is placed on a computational entity, whether self-like or something else, where we have no way of assessing the plausibility that such an entity could exist. It is to avoid that trap that I have chosen here to focus, where possible, on the SR option, which is arguably the more informative one from an analytical perspective in any case.

A final point, specific to the modular/constitutive model, is the neuroanatomical distinction this implies, that there should be two categories of conscious percepts. The first of these, referred to here as primary percepts, would be those for which direct sensory input is instructive in specifying which modules are to be activated irrespective of modulatory or permissive inputs originating from other sources, including higher-order integrative processes. Secondary percepts are those initiated by output from those higher-order processes, whether cortical or cortico-thalamic in origin, where it is their output that acts in an instructive fashion. This highlights the differing roles played by inputs to neuro-computational processes compared to their outputs, a key point when it comes to constructing a minimal model of the functions performed by a non-integrative consciousness (see section 6). The more usual distinction in phenomenological terms is between phenomenal and access consciousness, but there is a degree of category overlap: that phenomenal, anoetic experiential states are primary percepts in some instances but not all, whereas access consciousness, including all noetic and autonoetic states, consists by definition only of secondary percepts.

## 3 A modular/constitutive model of consciousness as it might apply to vision

The core premise of this paper is that the contents of consciousness can be generated by modular structures, small groups of neurons in other words, each capable of producing a particular sensation when instructed to do so. Zeki (2003) has suggested a modular model for vision to explain certain subcomponents of the visual experience, but the idea can be extended to account for the construction of the visual display itself by adopting something closer to the hotspot idea used by Berridge and Kringelbach (2015) to account for the subcortical production of sensations of pleasure, of “liking” rather than wanting. The visual display could then be constructed point-by-point by the action of separate modules, each acting as a hotspot. While this differs from the majority view of how conscious percepts are generated, it is not difficult to understand in conceptual terms. The conceptual problem arises if we choose also to assume, as I do here, that the causal effects of being aware of an experiential state are also localized, to the immediate vicinity of the module that produced it. This challenges not only our intuition, that consciousness operates globally, but also the consensus view, that it is an integrative construct that arises from the activity of highly interconnected cortical networks responsive to conscious signals (Varela et al., 2001; Sauseng and Klimesch, 2008; Petersen and Sporns, 2015). However, despite widespread acceptance of the integrative model, there is no scientific justification for rejecting the alternative, that conscious sensations can be localized in both their mode of production and their ability to exert causal effects. The reasons why are dealt with in the paper preceding this one (see Lacalli, 2025), the point being that the hard problem prevents us from knowing how our global sense of awareness is generated at a mechanistic level. Being aware of individual sensations may then simply be a matter of those sensations having been produced, regardless of whether the distance over which they are able to exert direct causal effects is large or small^3^.

To proceed further along this line of argument requires the additional assumption that conscious visual experience has the property of position-dependence, meaning that the spatial position of each module, either in absolute or relative terms, has perceptual consequences. The remainder of this section is devoted to exploring this idea further, but it is in my view more than an idea, but instead very possibly the only idea offering a plausible mechanistic explanation for how patterns of sensory input and processing can be mapped from the neural substrate to a corresponding perceptual space, whether for vision or for any other sensory modality. For those who might disagree, I challenge them to offer alternative suggestions, specific as to mechanism, as to how the trick might otherwise be done. A complication for conscious vision is whether it incorporates integrative features, as the binding argument would suggest (see Feldman, 2012; Lo and Zeki, 2014; Zeki, 2020; Peters et al., 2024; Di Lollo, 2012 for the counterargument), which would make the visual display we experience something of a hybrid, combining features of both primary and secondary percepts. This is less of a problem for this analysis than one might suppose because my explanatory target is the structure of the visual display in and of itself, which can be treated as a primary percept so long as the integrative features of conscious vision, whatever those are, are later evolutionary additions.

One reason for using vision as an example is the central role it plays in behavioral control, to quote Merker (2012, pg. 48), that it is “the dominant sense in terrestrial vertebrates and the general framework with which the other senses are aligned.” While this may be something of an over-generalization, given the role other sensory modalities play in the lives of various species, notably nocturnal ones, the importance of vision to species active during daylight can hardly be denied. Vision in vertebrates dates to the Palaeozoic, and well-developed eyes are in fact a key feature for distinguishing fossil vertebrates from non-vertebrate chordates (Shimeld and Holland, 2000; Lacalli, 2001). Consciousness in vertebrates, in contrast, is generally assumed to track the evolution of the neocortex, which would place its origins near the base of the amniotes, among early terrestrial vertebrates (Cabanac et al., 2009; Allen and Trestman, 2020). If we accept both that scenario and Ordovician conodonts as a model for ancestral fishes, there would have been a considerable interval when early vertebrates would have foraged and hunted prey visually, directing the eyes, head and body entirely without consciousness. Which then begs the question, that if a fully conscious visual display is so central to behavioral control in terrestrial vertebrates, how could their aquatic ancestors have survived without it? This deserves some further comment because it bears on the habits and survival strategies of early fishes. The key point is that their brains would not have been much larger than those of the protochordates that preceded them except for the addition of dedicated centers for processing sensory inputs, from the visual, olfactory and vestibular organs. Visual information at this early stage of vertebrate evolution would have been mapped topographically to the midbrain tectum, and then accessed to monitor changes in the animal’s surroundings due to its own movements, the motion of nearby conspecifics, and of moving targets that might be food items (Burr and Thompson, 2011). All of this, assuming it was accomplished by specialized computational filters, would have required a dedicated set of circuits able to perform the appropriate computational tasks with optimal efficiency.

Judging by the role vision plays today in predator/prey interactions, minimizing the time required for visual inputs to influence such behaviors has probably always been crucial to survival. Consider a shark attacking a school of anchovies for example. For the anchovies the response is schooling behavior, which is predominantly guided by visual cues (Viscido et al., 2004), but is optimally effective only if the response of the school as a coordinated whole is faster than the predator’s ability to adjust its own attack speed and direction. The implication, assuming this represents a long-standing adaptive issue, is that there has, since the Cambrian, been an ongoing arms race in mechanisms for visual attack and visually-controlled avoidance responses where the disadvantages of relying on a conscious display format for vision, given the time required to assemble and respond to such a display (Zeki, 2020), might have outweighed the advantages. The issue is not then whether fishes today do or do not have conscious vision, but rather with the case to be made for an ancestral mode of visual processing comprised of multiple computational filters, each able to respond selectively and as rapidly as possible to particular patterns of tectal activity.

It is a then separate question whether any one of those filters performed something akin to a global surveillance function. If so, it would have been that filter the conversion option would have converted to a conscious mode of operation. Given the range of tasks vision performs (e.g., see Frisby and Stone, 2010; DiCarlo et al., 2012; Poggio and Serre, 2013), this begs the question of why that particular conversion was an adaptive solution if, in consequence, the behavioral role played by all other filters was thereby reduced. The problem in part lies in our inability to judge the costs and benefits of different combinations of filters, some operating in a conscious mode and others unconsciously, but this difficulty extends to other sensory modalities. For those represented by phenomenal contents the issue is the hard problem, which denies us knowledge of how a conscious phenomenal state and its non-conscious counterpart differ at a mechanistic level. Arguments in principle are therefore not possible, because we cannot claim that there is necessarily a mechanistic difference between the two. Language and thought are more easily dealt with because of the evolutionary sequence: that if the ability to consciously perceive sound evolved before speech and language, which is almost certainly the case, speech and language would have been secondary additions to an already well-established auditory consciousness. There would then be no reason to evolve a zombie counterpart of an inner voice in competition with a conscious one already in operation. For vision, however, this argument does not apply because there is no basis for supposing that the ability to consciously perceive light evolved before the events that made a 2-dimensional visual percept conscious. It could instead have been a case of coevolution, with a pre-existing zombie form of vision being converted to a conscious mode of operation as the ability to consciously perceive light evolved. Hence the conversion option remains on the table which, for a modular/constitutive model of conscious vision, would mean that the relevant modular structure was already in place, operating in a non-conscious mode.

To ground the argument further in some particulars, consider a representational theory (see Gennaro, 2018 on representational theories) where the conversion option would simply convert unconscious representations directly into conscious ones. Since one function of conscious vision is to provide a searchable visual database, we would then require a preconscious visual representation that was searchable, but also a preconscious mechanism for performing the search. If the latter depended on a self-like entity, it is the output of that entity that would, with conversion, be rendered conscious. This accords with the idea that it is the interaction between sensory representations and a self that generates an experiential state (e.g., see Merker, 2013), but if a zombie self is also possible, we have no more understanding of the adaptive advantage of converting its mode of operation to a conscious one than we do for any other such conversion. What is required instead is a reason that zombie vision and conscious vision are mechanistically different, as that is the condition that must be satisfied if there are to be circumstances where the costs and benefits favor one over the other. This condition would be guaranteed for modular/constitutive model if the visual percept were grounded in real 3-dimensional space by a property, position-dependence, that is intrinsic to awareness and has no computational counterpart, which would break the cycle in Figure 1 for both conversion and reallocation. By doing so it would move the argument one step beyond simply asserting that, having evolved, consciousness must in principle have some advantage, because for conscious vision the source of the advantage could be identified: that it derives from the property of position-dependence and the link between real space and perceptual space that property makes possible.

There are two problems here, the first being whether we can assert that there must be a mechanistic difference between conscious vision and all possible non-conscious alternatives, or if it is at best a conjecture. Logic would suggest the former: that what we are now asking of zombie vision is more than the production of a non-conscious phenomenal state, but instead of a mapping generated computationally that embodies spatial information that is made available to the neurons that monitor the map by mechanisms other than computational ones. This is a contradiction in terms if there is no way for this to be accomplished except through consciousness, and indeed there is the potential here for an operational definition of consciousness based on access to information embodied in exactly such a map. But without knowing how conscious visual percepts are both generated and monitored at a mechanistic level, the possibility of valid counterarguments to the above claim cannot be ruled out. Hence I will treat the non-existence of a zombie alternative to a position-dependent conscious visual percept as a conjecture, albeit one with strong logical support, which makes the conclusions of this account, in that respect, conditional. The second problem is that, even if the conjecture is correct, establishing an advantage in principle for conscious vision does not tell us precisely what that advantage is. The result of the entire exercise is then less than fully satisfying from an evolutionary perspective, but on the positive side the status of hard problem is changed, because it is shown to be less an absolute limit on our understanding of why the brain might not just as well operate in the dark, without consciousness, than an obstacle that can be overcome by a suitably chosen set of assumptions^4^.

**FIGURE 2:**
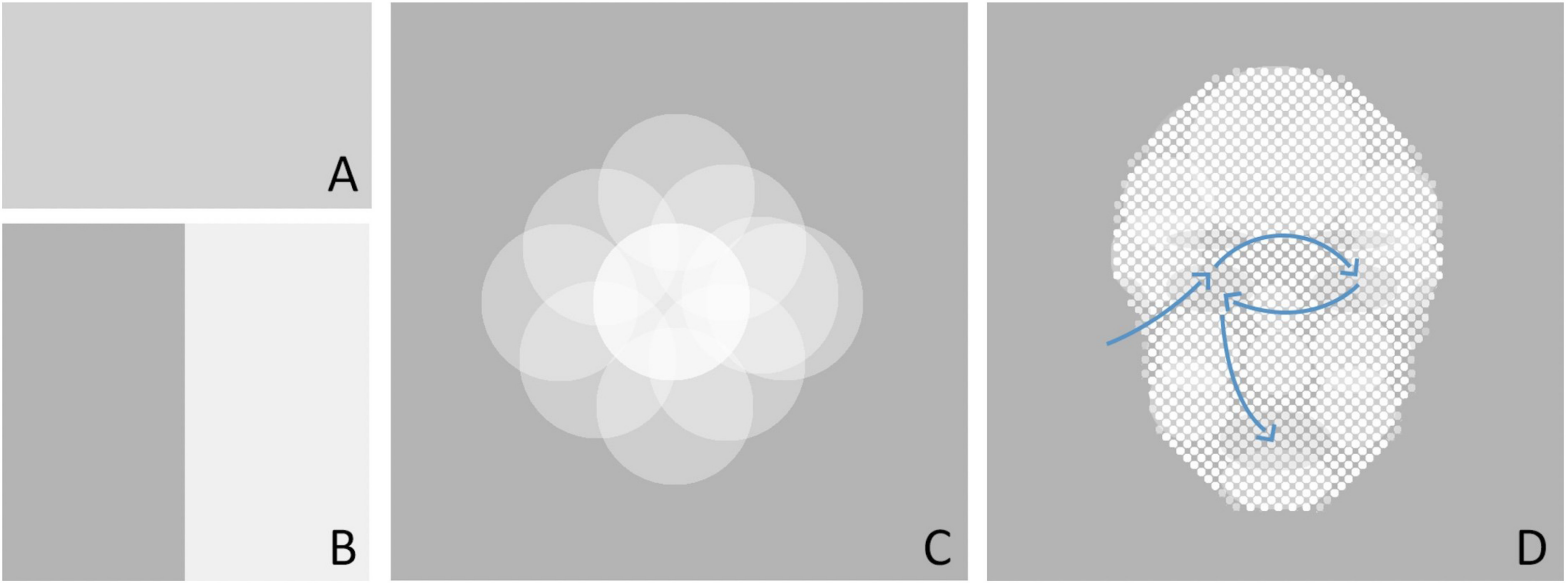
Stages by which a modular/constitutive visual percept might be constructed if its resolution was incrementally improved by the evolutionary expedient of adding more modules. **(A)** Conscious vision in the absence of position-dependence where the visual percept simply averages the input from both eyes. **(B)** Conscious vision dependent on two modules, one for each eye, so the percept distinguishes between inputs to the left eye, shaded in this case, compared to the right eye. **(C)** Multiple modules, which allows the percept to display a rudimentary image, in this case of a light source toward which gaze is directed. The contribution of each active module to the percept is shown as having well-defined edges, simply as a matter of convenience, where they might instead have been realized as a set of focal centers whose amplitude grades off over distance. Overlap is possible, as in panel **(C)**, if the modules are not structured and positioned to avoid this outcome. **(D)** A more fully evolved visual system with many more modules, the majority of which are now much smaller (shown where active as white dots) and form a non-overlapping array, here imaging a face. The limited resolution is intended to show how the face might appear at an intermediate stage in the maturation of the visual system, but it could also represent an intermediate stage in an evolutionary process. Blue arrows show successive steps in gaze during a hypothetical conscious visual search, the question being whether these can be supposed to have different costs and benefits compared with gaze shifts under the control of computational filters.

Setting aside the above conceptual issues, along with the various caveats concerning conversion, I want to move on to the problem posed by the reallocation option, of evolving conscious vision from the ground up. Multiple evolutionary innovations are required with this option because it depends on new structures (the modules) performing new functions. There is first the problem of devising a mechanism for generating a conscious awareness of light sensations regardless of the source of the stimulus. The resulting percept would then do no more than sum the inputs from both eyes, so if one is brightly illuminated and the other is shaded, the resulting percept would be uniform and intermediate in intensity (Figure 2A). To provide even the most rudimentary information about stimulus source we would need, at the very least, a means of distinguishing inputs to each eye. But the ability to do so (Figure 2B) is a key step toward generating a full visual display because all that requires is more modules, each producing an output reflecting its topographical location (Figure 2C). The resulting percept would automatically manifest as a 2-dimensional display if it exists to direct gaze, because eyes have two orthogonal degrees of freedom, up/down and side-to-side, which by default places the viewpoint between the eyes at the point where those axes converge. The problem is how to evolve a conscious mechanism of gaze control when a highly effective unconscious one with multiple capabilities (e.g., see Schiller and Tehovnik, 2005; Hutton, 2008) is already in place. This requires a balance to be struck between conscious gaze control and the competing actions of unconscious mechanisms, which might, for example, depend on evolving a means of preventing gaze from being unconsciously re-directed from a percept that deserves more attention than a computational filter would give it. An additional source of information would be required to justify conscious intervention, which is where encoded memories enter, as a way of providing that information. A competition between mechanisms is the predicted result, evidence for which comes from the different timescales of gaze shifts during visual search of faces (Kauffman et al., 2019; Taylor et al., 2024), the problem being that we do not know how these timescales map to underlying mechanisms, whether conscious or not. Caveats aside, the point is that once a conscious component is present, the unconscious process of registering visual stimuli, seeing in other words, would for some subjects be replaced by a process dependent on consciousness that I will refer to as “looking” that through memory is adjustable in response to past conscious experience.

But if looking requires agency, and agency must be learned, what we also require is a learning process that occurs in real time and is reinforced by a reward. This accords with the idea that consciousness must be learned (Cleeremans, 2011; Cleeremans et al., 2020; Lacalli, 2024a) and with theories placing associative learning at the core of what consciousness achieves (e.g., Ginsburg and Jablonka, 2007; Jablonka and Ginsburg, 2022). Both depend on inputs from memory and imply knock-on effects on the way memories are encoded. Precisely why is shown in Figure 2D, a representation of a face as it might be consciously perceived by a neonate. I portray the image as poorly resolved because for our species vision develops incrementally after birth (Colombo, 2001), an indication that one or more steps in the processing sequence are operating in a less than optimal way (Kiorpes, 2016). This is considered adaptive because it permits a cognitive transition in the way visual features are categorized, from the general to the specific (Vogelsang et al., 2018). Early memory encodings would then be overwritten as details were added to templates established earlier in the learning process, where it may matter whether those templates were generated in the first instance by conscious visual inputs or unconscious ones.

The idea that gaze control and selective attention are core functions of conscious vision accords with the widespread recognition of the adaptive benefit of controlling attention, both in general terms and for vision (Thompson and Parasuraman, 2012; Moore and Zirnsak, 2017; Pitts et al., 2018), but in particular that it is “through [gaze shifts] that the brain most immediately expresses its behavioral priority of the moment, and it is to them that it yokes the recruitment of the remainder of its motoric equipment in implementing actions” (Merker, 2012, pg. 46). The importance to this conception of having a central viewpoint prompts me to suggest a way of assessing theories of consciousness (at least as they apply to conscious vision as we experience it), which is that any theory should be required to explain both how a conscious visual display like our own is produced and also how it is anchored in the real world by its viewpoint. One problem with connectomal network-based theories is their failure to provide the bridge between physical space and perceptual space this would appear to require, a problem easily solved by a modular/constitutive model. Which then directs attention away from the cortical integrative processes on which network-based theories depend toward the subcortical neural centers Merker identified as important including, for vision, the dorsal pulvinar. There is a caveat to consider as well, however, that it again matters whether conscious vision evolved by conversion or reallocation. For the former, insights into the structure of the visual display will inform both our understanding of the conscious percept and the unconscious computational processes on whose conversion to consciousness that percept depends whereas, for reallocation, there is no such linkage. Even a complete understanding of the neural circuitry responsible for unconscious responses to visual stimuli in brains like ours would not only then be inadequate as a basis for explaining conscious vision, but might well be entirely irrelevant to that explanation.

## 4 What does it mean to say that “consciousness must be learned”?

**FIGURE 3:**
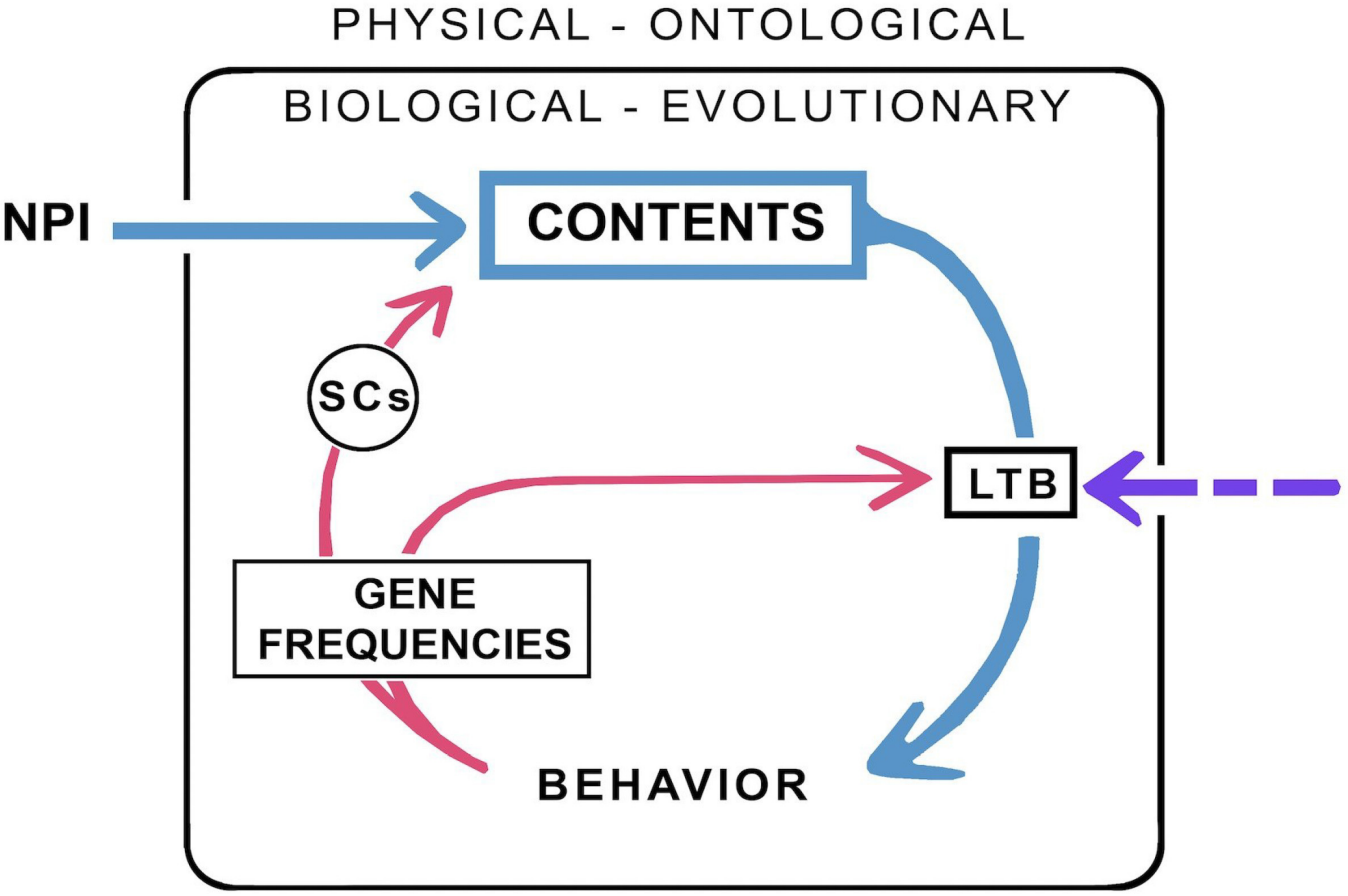
The consciousness machine, a device for thinking about how consciousness evolved, modified from Lacalli (2023). Each cycle represents the transition from generation to generation, so that contents change incrementally in consequence of the actions of natural selection. The outer casing encloses all the algorithmic processes carried out by the brain, which may or may not depend on external inputs. The latter are required for neurophysical models of consciousness, both to supply a physical source, in the form of a neurophysical input (NPI, blue arrow), from which the various contents of consciousness are constructed, but also to establish a link to behavior (LTB). A second external input (the dashed purple arrow) is required to ensure that conscious sensations are not epiphenomenal, a condition that must apply to both a hypothetical signal and conscious awareness of that signal. Because the machine operates in evolutionary time, real time events are not represented, so the internal workings of the box labeled LTB are invisible to evolution. This highlights the distinction that needs to be drawn between the link to behavior as a guarantor of causal efficacy and the learning process required in each generation to generate conscious agency, the latter then being an externality that must be dealt with separately. SCs are the selector circuits that specify which sensations are to be evoked, a requirement for any theory where consciousness depends on neurocircuitry.

Learning and memory figure in a number of theories of consciousness, but it is not always clear what is meant by such statements as “consciousness must be learned” (Cleeremans, 2011), “the evolution of minimal consciousness was driven by the evolution of learning” (Jablonka and Ginsburg, 2022) or, to bring in memory, that “all theories of consciousness must at some level be memory theories of consciousness” (Lacalli, 2024b). Conscious vision is then a useful test case for clarifying how these statements should be interpreted. The first point to make concerns time scales and applies to any theory that incorporates evolution: that care is required to separate events lodged in evolutionary time from those that occur in real time. My preferred way of dealing with the problem involves the conceptual device that I call a consciousness machine, an example of which is shown in Figure 3. The machine operates in evolutionary time and requires two things for consciousness to evolve: (1) a source of conscious sensations that, for a physicalist model, depends on inputs from the ontological realm (the blue arrow) and (2) a link between the emergent contents of an evolving consciousness and behavior (the box labeled LTB, the link to behavior). The latter is required so that the advantages of consciousness, whatever those are, generate a positive feedback loop that drives further evolutionary change. What the figure does not show are the processes internal to the LTB, which is literally then, from an evolutionary perspective, a black box. Theory tells us, however, that the LTB must incorporate a real-time memory-dependent learning process if a form of consciousness is to emerge that confers agency on the individual [see Lacalli (2023, 2024a) for the full argument]. The evolution of consciousness will thus be accompanied of necessity by the emergence of conscious skills that can be learned, where understanding what this entails requires a shift in focus to real-time events. Yet those events too will have an ontological component (the dashed purple arrow) if we require, as I do here for vision, through position-dependence, that there is an absolute dependence on a property that is ontological in origin.

So, taking vision again as an example, the evolutionary starting point would be an emergent visual percept, faint perhaps in the first stages of this process, but able either to direct gaze to a particular point in 3-dimensional space or sustain it there in the face of unconscious mechanisms that would shift it elsewhere. There would then have been a competition in real time between emergent conscious mechanisms of gaze control and reflexive mechanisms already in place, resulting in evolutionary time in the progressive emergence of a division of labor between the two depending on their relative adaptive benefits. This might depend on differences in efficiency, that conscious vision might, for example, be more energetically efficient in performing some tasks and so would subordinate unconscious mechanisms for those tasks. Or, it might be that visual memories encoded through a conscious mechanism incorporate meaning in a way that zombie vision cannot, allowing the resultant memories to better reflect the benefits and hazards of the real world. Meaning by most definitions is assumed to depend on an experiential component conferring valence (Johnson, 2017; Barsalou, 2020; Shapiro and Spaulding, 2024), which would enter the learning process in the reinforcement step. However, if a zombie counterpart of valence is possible, able to perform the reinforcement task just as effectively, then a form of consciousness without meaning would also be possible. This complication is taken up in more detail in the next section, whereas here the issue I want to address is whether there are sensory modalities other than vision that might be position-dependent and hence able to drive the learning process just as effectively. Somatosensory experience is an obvious candidate because it has a directional component, but so does any content involved in phasic orienting that is routed through the intermediate layers of the colliculus (see Merker (2007), pp. 67–68 for the full argument). The complexity of the motor tasks involved, whether this involves reorienting the head or more complex kinds of gestural control, of reaching for or grasping objects perceived, might then be simpler than for visual gaze control, but the evolutionary issues are the same if we think about the nature of the percept in each case as it first evolved: that vision as we experience it is highly resolved and rich in information, but this would not have been the case if it began as a simple percept as shown in Figure 2B. The relevant motor tasks would then have been no more demanding than the simplest of somatosensory responses, of discriminating between left and right. There is also the caveat that not all species rely on vision to the same extent, that some have poor vision or none at all, the blind mole rat being an example, and members of our own species are born blind, yet none of these situations results in an impairment of conscious agency. This argues against vision being solely responsible in any species for the learning process through which agency is acquired, but also against the balance between conscious and reflexive mechanisms for gaze control being the same across species. Extended to conscious agency more generally, the implication is that agency should vary across species in being limited to tasks specifically learned during the developmental period during which the neural pathways controlling behavior become fully operational irrespective of the sensory modality, or modalities, involved. So, for early mammals, especially nocturnal ones living in burrows, tactile skills would have been at least as important if not more so than visual ones, implying a learning process that may have been predominantly somatosensory.

To conclude this section, I want to return to the problem of the role visual memory plays in the construction of a cognitive model of 3-dimensional realty. The latter depends on the ability to assess object size, spatial position, and the 3-dimensional character of identifiable objects viewed from different distances and angles (Linton, 2022), where it may be the record of gaze shifts that provides the oculomotor maps on which these abilities depend (Hannula et al., 2010; Zimmerman and Lappe, 2016; Ryan and Shen, 2020; Wolff and Lappe, 2021). If visual memories encoded consciously as gaze shifts were able to perform this role in a way that computational filters cannot, that in and of itself could explain the adaptive advantage of conscious vision. To test this as a hypothesis we would need to know more than we currently do about visual memory, and specifically the role played by gaze shifts generated by visual searches, but two potential benefits of conscious vision are: (1) that conscious encodings are different because they map to templates established by conscious gaze shifts in the earliest stages of visual memory formation that would not otherwise exist, or (2) that different value is assigned to conscious versus unconscious encodings through valence. The point here is that the acquired skill of conscious “looking,” whether accompanied by conscious valence or not, is different than “seeing,” if seeing is defined as relying entirely on computational filters. This is encapsulated in the idea that the advantage of conscious vision derives from relying on gaze control informed by conscious cues in a learning process that combines attention control (AC) and memory (AM) as equal partners in the enterprise: hence an ACAM model of conscious learning where the details of what M refers to, i.e., the precise memory systems involved, is not specified. Nor does it matter for this analysis. And, if looking incorporates meaning, the learning process by which agency is acquired would begin the process by which an anoetic form of conscious vision acquires a noetic component, which can be thought of both as an evolutionary innovation and, for an individual of our species, a natural part of post-natal visual and behavioral development.

## 5 Evolutionary sequence and codependence

This section addresses the question of how the contents of consciousness will have changed over time. The typical trajectory for any evolutionary innovation is to go from simple beginnings to something more complex, but it is not immediately evident what, for consciousness, is simple and what is complex. Simple might mean few contents and complex many, so the sequence would be one that added to an initial core of one or a few contents. Or, there might have been multiple contents from the start, but instead of those we experience today, a set of ur-experiences from which our more fully-differentiated form of consciousness has been derived. Since I have no way of choosing between the various possibilities, I will start by making an argument in principle based on two points, that (1) consciousness only enables individual agency through a learning process, and that (2) formats, of which vision is an example, are more useful than qualia for understanding what that learning process entails. For vision, the argument from the previous section is that we must learn to look, whereas no comparable learning process is required for phenomenal experience such as hearing a sound, feeling pain or perceiving light. This implies that if consciousness evolved only because something was being learned in each generation as it did so, the first content, if there was only one, would have been a format. But formats cannot exist without the qualia from which they are constructed, so that first content would have been a format coevolving with the quale it employs, which is different from supposing that a quale (light for example) evolved first and the format (vision) afterward.

**FIGURE 4:**
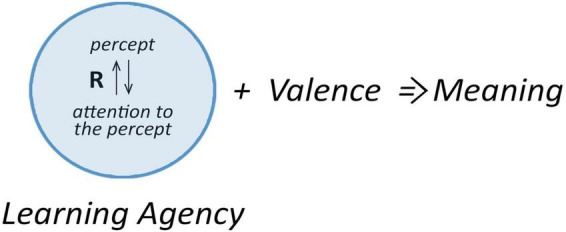
Separating agency from meaning. For consciousness with agency to evolve, using a position-dependent modality such as vision as an example, we require a learning process (circumscribed by the blue circle) such that attention is selectively directed to a percept which, for a modular/constitutive model of conscious vision, would be position-dependent. The reinforcement step required for the learning process (R) can then be either conscious or unconscious. The figure shows the situation if R is unconscious, which would be the case if unconscious valence-like phenomenal states can exist as a consequence of neural activity. For consciousness to incorporate meaning, a separate step extrinsic to the learning process would then be required to incorporate valence and hence meaning, in which case position-dependent contents could evolve independently of those conferring valence. The alternative is for valence to be intrinsic to the reinforcement process (R would depend on experiential states), so agency and meaning would be acquired together. At issue here is the question of whether consciousness could evolve without meaning, so that a consciousness consisting of only one content, say only vision, would be possible in principle.

Next, to take a minimalist stance, consider an emergent consciousness consisting of vision alone, one format in other words, where its advantage over zombie vision depends on awareness of visual stimuli being position-dependent. The question then is whether this is plausible as a hypothesis or, to generalize this claim, that a form of consciousness could have evolved consisting only of contents that are position-dependent. That more general claim avoids the problem referred to in the previous section, of relying on vision alone for learning agency, but neither option addresses the question of how to deal with contents that are not position-dependent, including contents conferring valence. For those the zombie argument applies, that without a clear understanding of the mechanistic differences between a conscious experience and a corresponding non-conscious phenomenal state, there is no justification for supposing that there can be conditions where the conscious option is more adaptive. Referring to Figure 4, the key issue with respect to evolutionary sequence is whether conscious inputs conferring valence act inside or outside the circle representing the learning process. If inside, those inputs would be available to provide the reinforcement needed for learning to occur, adding a synergy, so learning and meaning would be acquired together. In contrast, if the reinforcement process is just as effective when it depends on a non-conscious phenomenal state, then agency is acquired, but the actions involved are carried out without being accompanied by meaning, or at least meaning as conferred by consciousness. The distinction I am making here, between contents that are position-dependent and those that are not, maps approximately to the difference pointed out by Godfrey-Smith (2019) among others, between contents responsible for percepts representing the objective world, external to the animal, and those required for evaluating those percepts, i.e., affect or feelings. Since these operate in a synergistic way in our own consciousness, the expectation is that an emergent consciousness would contain both, which accords with the argument that affect should lie at the core of an evolved consciousness (e.g., see Cabanac, 1992; Dickinson and Balleine, 2010; Butlin, 2020; Cleeremans and Tallon-Baudry, 2022). Contents conferring valence should therefore have been there from start, but we have no explanation for why if there are no mechanistic differences between the way those experiential states are produced and responded to compared with their hypothetical zombie counterparts.

There are, in sum, three options to consider: that (1) consciousness as it first evolved consisted of multiple contents, some position-dependent and others conferring valence, or (2) that it consisted only of position-dependent contents or (3) only of contents conferring valence. The first of these options yields a consciousness that incorporates both agency and meaning, the second a consciousness that confers agency without meaning, and the third, assuming that behavioral responses to contents conferring valence are not learned responses, of meaning without agency. And, though both (2) and (3) fall short of the kind of consciousness we experience, this does not preclude them being part of the sequence by which a form of consciousness combining agency and meaning actually evolved. Option 3, for example, could be an intermediate in this process, but only if the adaptive advantage of consciousness derives from something other than the exercise of conscious agency. Conscious valence might, for example, reduce connection costs for a broadcast-based workspace function, which for a non-integrative model of consciousness would act locally rather than globally. But this is no more than a conjecture, and brings us no closer than before to an understanding of whether there is something inherently more “meaningful” about memory encoding accompanied by conscious valence compared with the same encoding accompanied by a corresponding non-conscious phenomenal state.

Having reached what appears to be a point of diminishing returns in the narrative using functional arguments alone, a change in focus is in order, to arguments grounded more in ontological and evolutionary considerations. I will deal with evolutionary issues first, which requires attention to the ancestral condition, of a consciousness consisting of ur-contents that may have been quite different from those characterizing our consciousness today. The ur-contents of an ancestral consciousness might, for example, have been less well-differentiated than they are in our consciousness, so that experiences that for us represent separate sensory modalities could have been experienced together, as a combination of subcomponents not yet rendered fully distinguishable by natural selection (see Lacalli, 2022 for the full argument). But if we can suppose that at least one ur-content was position-dependent, that should justify both its evolution and the supposition that the quale representing it was optimized for the function it performed. In other words, it is entirely plausible that an ancestral form of conscious vision would have utilized a quale we would recognize as light-like if that was the most adaptive way, or perhaps the only way, of constructing a position-dependent visual display. This would then affect the character of other emergent qualia, because selection would eliminate any light-like character from those in order to minimize interference with conscious vision. But by the same token those other ur-qualia would need at the very least to be distinguishable from each other to differentiate them in their role as contributors to the reinforcement process by which visual agency is learned. On this basis one could argue that ur-qualia representing sensory modalities that were not position-dependent would have been less constrained in the form they took compared with qualia adapted to suit particular position-dependent functions. That lesser degree of constraint would then be reflected in the character of the descendants of those other ur-qualia today.

This leaves unanswered the question of why experiential states that are not position-dependent would be conscious at all. One answer is that this could be due to evolutionary descent, homology in other words, because conscious contents that are not position-dependent could nevertheless have been derived by descent from ur-experiences that were. All of the descendant contents of those ur-experiences would then be conscious by virtue of being homologous as experiences, providing an evolutionary basis for the common currency argument: that the currency of exchange among different forms of conscious experience has always been common because they have a common origin. An emergent form of conscious vision would then have had a valence-dependent component right from the start, with meaning conferred on visual experience in a way that no zombie form of vision could. This begs the question of whether there is evidence for conscious contents that today combine position-dependence and valence together. Pain in its various manifestations is one candidate since, depending on circumstance, it can be localized, through referral, but also has affect-like characteristics (Tsai et al., 2019). I would also cite here the observations of McGlone et al. (2014) on hedonic coupling, that two qualia (touch and pleasure in their example) appear to be linked in ways that could imply homology. There is an equally plausible counterargument that the link may simply be that the two are evoked together by the same stimulus, but it is nevertheless a useful exercise to seek out such linkages, as evidence for an especially close relationship between different categories of qualia potentially indicative of homology.

BOX 1 **Physical explanation as constraints on mechanisms.**

In physics, to say that an experiential state “has properties” is not (at least initially) a statement about meaning or content, but about what a physical mechanism must supply to be explanatory. Any mechanism that acts causally in real space and time must, at minimum, be describable in terms of identifiable degrees of freedom (state variables), lawful dynamics, and constraints (including symmetries). These ingredients delimit what the mechanism can do and how it responds to perturbations, and therefore define what kinds of outcomes can be generated reliably.

From this standpoint, the internal complexity of a module and the role of “integration” should be distinguished. In many biological systems, integrative processes do not encode content or act as centralized controllers, but instead coordinate outcomes by imposing constraints – for example by limiting degrees of freedom, interaction range, cross-talk, or geometry – thereby shaping which dynamics are accessible.

Vision provides a stringent test case because it imposes geometric constraints: perceptual space preserves structured relations in physical space, so any candidate mechanism must reproduce and stabilize this mapping. Affective states, by contrast, are not obviously governed by such spatial organization, suggesting that they may rely on different effective state spaces and constraint structures.

Finally, concepts such as “information” and “computation” are useful as descriptive tools – for example, Shannon measures can quantify precision and reliability – but they should not be treated as causal agents. Causal explanation resides in physical variables, dynamics, couplings, and constraints; information-theoretic quantities characterize performance once mechanisms are specified.

Moving from evolution to ontology, the question I would ask is how a physicist would respond to the statement that awareness must have properties. Assuming this was acceptable as a valid statement about one experiential state, light perception in the case of vision, the follow-up question would be whether all such states, as members of a definable category, can be supposed also to have properties, and if so what those might be. To clarify what would count, from a physics standpoint, as a mechanistically grounded explanation, I have sought input (see Acknowledgments) on the minimal physical requirements such an explanation would need to satisfy. Box 1 summarizes these, from which I conclude that the theory in question must first pass the test of being causally valid (the condition of causal closure to be more precise), but once it has done so, the existence of one property implies there may be others so long as there are multiple ways of constraining solutions to the relevant equations of state, through boundary conditions and the like. Properties other than position-dependence should then be possible, which incidentally, in evolutionary terms, would make the resulting experiential states homologous at a mechanistic level. I admit that at this point in time “having a property” is no more than a placeholder in the argument, representing something that we do not as yet fully understand. But that placeholder must nevertheless satisfy the conditions specified in Box 1, where I am here proposing position-dependence as the candidate property that makes it possible to formulate the argument in mechanistic terms. This resonates with Chalmers’ position as expressed in the conclusion of his 1995 paper: that consciousness is simply another kind of ontological phenomenon obeying a set of rules that need not accord with our preconceptions concerning causality as we experience it in our everyday lives. But we know this, or in any case physicists know this after a century of using quantum theory to explain the behavior of elementary particles that violate common-sense notions of causality at virtually every turn (Carroll, 2025; Gibney, 2025). This leads me to question whether the mysteries of consciousness are any greater, or different in kind, than those that confront us in any attempt to fully comprehend physical reality at a foundational level.

## 6 Conclusions, and the non-integrative minimum

Identifying the primary function consciousness performs amounts to answering the question posed by Chalmers (1995), of why the brain might not just as well operate in the dark, in subjective silence. This means discovering brain functions for which there is no zombie counterpart, either because no such counterpart could conceivably evolve, or if it could, that it would be less adaptive. None of these issues are easily resolved by arguments in principle except for the case of conscious vision, and then only if we adopt a modular/constitutive model that incorporates the provision that conscious awareness of visual stimuli possesses a property unique to the conscious condition. This property, position-dependence, has the potential (here treated as a conjecture) to act as guarantor of causal efficacy while being, at the same time, indispensable for the learning process on which conscious agency depends. If the conjecture is correct, the reason for a mechanistic difference between conscious vision and all possible zombie alternatives is made explicit: that the conscious control of gaze operates through a mechanism dependent on a visual percept that cannot exist except through consciousness. Conscious gaze control would then have different costs and benefits compared with all non-conscious alternatives, and circumstance would govern the evolutionary outcome of competition between them. The analysis is not designed to deal with species where vision plays a different, perhaps subordinate role in behavioral control compared with the human condition, nor do we know the sequence in which contents were incorporated into vertebrate consciousness as it evolved, where vision, despite its centrality to this analysis, may not have been among the first. But from the fact that some vertebrates have conscious vision we must conclude there was at least one function for which it provided an advantage. This could include the control of attention, memory encoding, the assignment of meaning to visual percepts, or some synergistic combination of these. Choosing among options is again a matter of conjecture, but there is a more general point to be made, that the explanatory target of the exercise has changed, from the question of why consciousness (conscious vision in this case) is adaptive to a narrower focus on the conditions under which conscious agency is more adaptive than its zombie counterpart. This redirects attention from consciousness as a phenomenon to the mechanism by which conscious agency is acquired, which is a matter of understanding the learning process on which this depends and the role that contents conferring valence play in that process.

**FIGURE 5:**
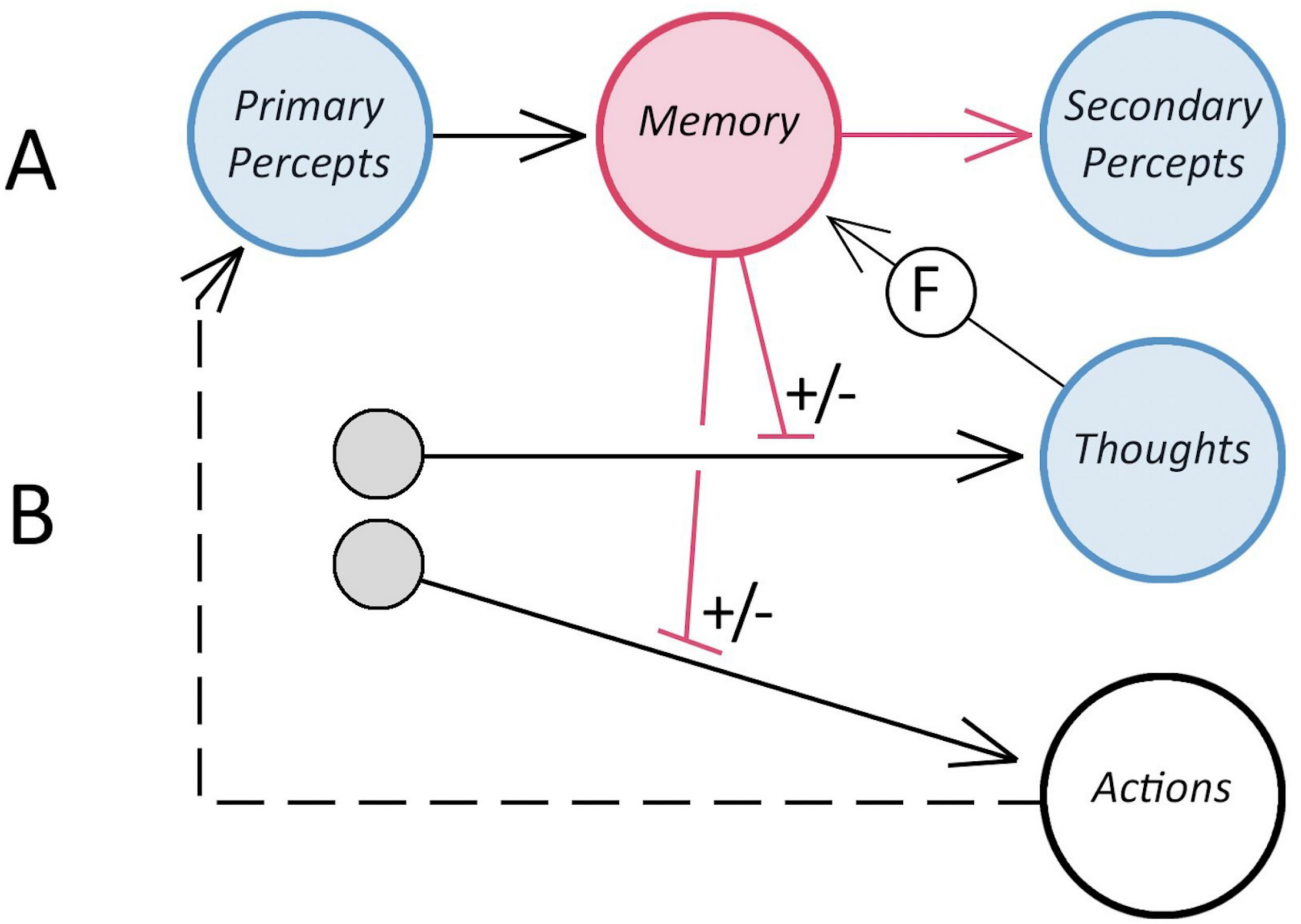
A proposal for how a modular, constitutive consciousness might operate in functional terms if, either because of limitations intrinsic to awareness or simply as a matter of evolutionary contingency, it does not participate in integrative pathways operating at scale. Conscious contents (in blue) would then act only indirectly on memory, actions, and secondary percepts, via non-conscious pathways (black arrows), because the modular/constitutive model limits their ability to exert causal effects over distance. Memory pathways, which also operate in a non-conscious mode, are in red. There are then two “streams” of consciousness. First **(A)**, a perceptual stream, of primary percepts that can be simple (phenomenal percepts) or complex (e.g., vision) that contribute to memory encodings which, when activated by recall mechanisms, generate secondary percepts including visual images and thought. Second **(B)**, the intervention streams, whereby conscious and unconscious memories influence actions in progress through motivational control mechanisms, altering an animal’s navigation through its habitat, for example [the MDIS model from Lacalli (2024b)], or for thought, navigation through a series of visual or linguistic states. Feedback is required for both of these, where for action sequences this would include external stimuli generated as a consequence of the action in question (dashed arrow), but for thoughts is internal (the arrow labeled F), whether initiated by an inner voice, visual images, or affective memories. The diagram accords with the premise, that from an evolutionary perspective consciousness should be thought of primarily as a servant of memory, performing a restricted set of functions that involve it far less in sensory integration than most theories of consciousness suppose. Missing from the figure is any explicit reference to attention control, despite its importance to this account, because it is a skill acquired through a developmental process, while the figure shows only causal structure, not specific real-time events.

Caveats aside, the picture of conscious vision that emerges from the assumption that it is modular and constitutive, the premise of this account, is of a dedicated mechanism for performing a specific set of visual tasks associated with attention control and memory encoding (the ACAM model), so it is only by acting through memory that conscious vision exerts a decisive influence on behavior. Conceptually this is very different from thinking of consciousness as either a unifying integrator of sensory processing or a source of global oversight in behavioral decision-making. For the latter, the question to ask in evolutionary terms, following Morsella et al. (2016, pg. 5) is “What is the most basic form of integration that requires consciousness?” But for the modular/constitutive model the question instead is “What is the minimum we require of consciousness if it is not integrative?” Taking this more limited view, Figure 5 shows a proposal for how such a consciousness might operate that accommodates the distinction introduced in section 2, between the role of primary percepts in memory encoding and the actions of those encodings as they direct progress through sequences of mental states that may be motivational (for action control) or linguistic (for thoughts). This accords with Pierson and Trout (2017), who identify volitional control of action as the core function of consciousness [similarly, the flexible response mechanism of Earl (2014)], but also with current ideas that see language as an extension of gestural control (Arbib, 2005; Gentilucci and Corballis, 2006) with a common syntactical structure (Pulvermüller, 2014; Boeckx and Fujita, 2014). It also clarifies the point made by Budson et al. (2022, pg. 270) that consciously we “perceive the world as a memory” as not being strictly correct for conceptual models that admit the possibility of primary percepts, but accurate otherwise as a statement about the role consciousness plays indirectly, through intervention, in the control of actions and thoughts. The modular/constitutive model is then useful for showing that there is an alternative to thinking about consciousness as an integrator first and foremost, making explicit the alternative: that it can be viewed instead as a servant of memory that need not participate in integrative processes to anywhere near the degree that most theories of consciousness suppose.

## Data Availability

The datasets presented in the study are included within the article, further inquiries can be directed to the corresponding author.

## Footnotes

1 The term consciousness will be used throughout in a general way, to refer to the ability to host and respond to any experiential state.

2 Note here that it is awareness that has properties not the signal that is its proximate cause, so awareness must incorporate some additional feature the signal does not possess that is accounted for by other means.

3 The hard problem enters this analysis in two ways that may appear incompatible but are not. First, as just mentioned, it prevents us from asserting that the subjective unity of consciousness requires an integrative mechanism, opening the way to a consideration of the modular/constitutive alternative. Second, in the event that the latter were to be proven correct, the limitations the hard problem imposes on our understanding of the nature of experiential states would also be open to reexamination, specifically as to whether in all cases it denies us knowledge of the physical properties on which those states depend.

4 A reminder is perhaps required, that none of these arguments would apply without additional caveats to theories where consciousness is neither modular nor constitutive.
